# Multipurpose Mobile Apps for Mental Health in Chinese App Stores: Content Analysis and Quality Evaluation

**DOI:** 10.2196/34054

**Published:** 2022-01-04

**Authors:** Xiaoqian Wu, Lin Xu, PengFei Li, TingTing Tang, Cheng Huang

**Affiliations:** 1 College of Medical Informatics Chongqing Medical University Chongqing China; 2 Medical Data Science Academy Chongqing Medical University Chongqing China; 3 School of Public Health Weifang Medical University Weifang China; 4 The Children’s Hospital of Chongqing Medical University Chongqing China

**Keywords:** mobile apps, app, mental health, mHealth, content analysis

## Abstract

**Background:**

Mental disorders impose varying degrees of burden on patients and their surroundings. However, people are reluctant to take the initiative to seek mental health services because of the uneven distribution of resources and stigmatization. Thus, mobile apps are considered an effective way to eliminate these obstacles and improve mental health awareness.

**Objective:**

This study aims to evaluate the quality, function, privacy measures, and evidence-based and professional background of multipurpose mental health apps in Chinese commercial app stores.

**Methods:**

A systematic search was conducted on iOS and Android platforms in China to identify multipurpose mental health apps. Two independent reviewers evaluated the identified mobile apps using the Mobile App Rating Scale (MARS). Each app was downloaded, and the general characteristics, privacy and security measures, development background, and functional characteristics of each app were evaluated.

**Results:**

A total of 40 apps were analyzed, of which 35 (87.5%) were developed by companies and 33 (82.5%) provided links to access the privacy policy; 21 (52.5%) apps did not mention the involvement of relevant professionals or the guidance of a scientific basis in the app development process. The main built-in functions of these apps include psychological education (38/40, 95%), self-assessment (34/40, 85%), and counseling (33/40, 82.5%). The overall quality average MARS score of the 40 apps was 3.54 (SD 0.39), and the total score was between 2.96 and 4.30. The total MARS score was significantly positively correlated with the scores of each subscale (r=0.62-0.88, *P*<.001). However, the user score of the app market was not significantly correlated with the total MARS score (r=0.17, *P*=.33).

**Conclusions:**

The quality of multipurpose mental health apps in China’s main app market is generally good. However, health professionals are less involved in the development of these apps, and the privacy protection policy of the apps also needs to be described in more detail. This study provides a reference for the development of multipurpose mental health apps.

## Introduction

Anxiety, depression, stress, and other mental health conditions are increasing worldwide, involving family, study, work, social intercourse, and other aspects. Nearly 1 billion people worldwide have been found to have mental disorders [1]. In China, the weighted lifetime prevalence of mental diseases, except dementia, among the population over the age of 18 years is as high as 16.57% [2]. Although mental disorders will impose varying degrees of burden on patients and their surroundings, only 15.7% of individuals with lifelong mental disorders in China seek help [3]. This situation is still obvious in cities with high economic development. A study in Shanghai, China, revealed that approximately 21.4% of the subjects reported depressive symptoms but only 4.7% sought mental health services [4]. The general underutilization of mental health services is worrying. The main reasons for this are the shortage of resources, the limited number of mental service professionals, and the uneven geographical distribution of mental health services in China [5]. Additionally, stigmatization of mental health services [6], low perceived demand [7], and economic constraints make people reluctant to take the initiative to accept mental health services [8].

Mobile apps are considered an effective way to eliminate these obstacles, improve mental health awareness [9], and contribute to symptom tracking and self-management [10]. Mobile apps are not limited to a particular time and place and realize the large-scale provision of cost-effective medical services, especially for people who find it difficult to receive traditional psychological services, for example, the population in rural areas with relatively low economic development and those who find it difficult to receive services face-to-face because of special reasons. During the COVID-19 outbreak, the spread and uncertainty of the pandemic caused a pessimistic mood of anxiety and fear for some patients, medical workers, and the general public [11]. This led to a sudden increase in mental health problems and their higher incidence rate, and mental health needs worldwide [12,13]. However, social and interpersonal networks were relatively closed because of epidemic prevention and control. Thus, traditional mental health services were difficult to obtain. Mental health apps can overcome the limitation of distance and expand the scope of psychological counseling for people. This also highlights the potential of digital health in improving the coverage of mental health services [14,15].

The advantages of mobile apps and the growth of mental health demand are making mental health and adaptive mobile health (mHealth) apps increasingly popular. Furthermore, there is an urgent need for more research to promote the formulation of better mental health service recommendations, especially in China’s huge untapped market [16]. The main categories of mental health apps are assessment, tracking or monitoring, treatment, and multipurpose [17]. However, the multipurpose mental health apps integrate evaluation, monitoring, treatment, and other mental health services into 1 platform to provide users with one-stop services. They are the most popular apps for all ages [17]. However, there is currently no specific evaluation for multipurpose mental health apps. One study referred to multipurpose mental health apps, but there is a lack of standardized measures to evaluate and compare the quality of apps [18]. A study searched and evaluated China’s mental health apps [19]. However, some features of the apps, such as the professional background of app development, the theme distribution of the function, and the user privacy protection policy are still unclear. The privacy protection of applications is an important reference for people to choose mental health apps [20], and the lack of a professional background in the process of app development may reduce users' confidence in the apps. Simultaneously, we found that great changes have taken place in China’s app market with the development of the internet and cell phone manufacturers. The app market developed by cell phone manufacturers replaced third-party app markets, such as 360 and Baidu, and occupied the main share of China’s app market, together with Tencent My App [21].

Therefore, this study aims to investigate the characteristics of multipurpose mental health apps in China, evaluate their quality, and describe the main functions, user privacy protection, and professional background of the development process of multipurpose mental health apps in the current market in order to help users make more informed choices and provide reliable evidence for app developers.

## Methods

### Systematic Search Strategy

This study featured a systematic search and content analysis of multipurpose apps on mental health in Chinese app stores on December 17, 2020. Huawei cell phones occupy the first place in the smartphone market share in mainland China [22], and the Huawei AppGallery has become the largest Android app store in China [21]. Moreover, Tencent My App, provided by the Chinese internet giant Tencent, is the second-largest Android app store in China [21]. Thus, we searched the Apple App Store (for iOS apps), Tencent My App, and Huawei AppGallery (for Android apps).

By preliminary test searches, the following keywords were determined: psychology, psychological counseling, psychological intervention, emotion, stress relief, anxiety, and depression. These keywords were searched anonymously using Chinese language terms in the app stores not logged into any user accounts. All search results were collected to ensure that all potentially relevant apps were captured. If an app exists in both iOS and Android and has the same design and content, the Android version was evaluated.

### Eligibility Criteria

After removing duplicates, each potentially suitable app was reviewed by 2 independent researchers based on the app name, screenshots, and description. In this round, apps were included if they (1) provide multiple mental health services, (2) focus on individual mental health consumers seeking professional help, and (3) are in the Chinese language. Apps were excluded if they (1) focus on content unrelated to mental health services, such as social and communication apps, e-books, heartbeat measurement, and pulse measurement; (2) target mental health service providers, such as doctors, nurses, or counselors; and (3) provide a single function only. All apps that met the inclusion criteria were downloaded onto test devices. Of these, apps were excluded if they are not usable because of technical errors or require special authentication (such as an enterprise or a school).

### Data Extraction

The relevant information provided by the app market was extracted to evaluate the descriptive features of the apps. The general characteristics of the apps, including platform, developer, target user, update time, star rating, and downloads, were recorded. Additionally, combined with the researchers’ use of the apps, the characteristics of the apps with regard to personal privacy protection and professional development background were extracted (Table 1). Furthermore, through a literature review and group discussion, we divided the main services provided by mental health apps in China into 6 categories: psychoeducation, counseling, self-assessment, question-and-answer (Q&A) module, stress relief, self-monitoring, and management. The characteristics of the apps and the main services provided were recorded by 2 independent researchers. All differences were resolved through discussion until the researchers agreed upon the results.

**Table 1:** General information collected for each app.

Assessment measure	Definition and values
Platform	Apple App Store, Huawei AppGallery, Tencent My App
Developer	Unknown, commercial, psychological service organization, individual developer
Target user	Children or adolescents, general public, specific for women and patients with mental health problems
Update time	Days from the retrieval date to the last update
Star rating	Star rating (out of 5) left by users in the app store
Downloads	Number of app downloads in the app store
Privacy protection	Does the description of the app claim to provide privacy protection? (Yes or no) Is there an obvious privacy protection logo in the process of using apps? (Yes or no) Does the app report relevant privacy protection regulations? (Yes or no)
Evidence-based and professional background	Does the app claim to be designed based on proven psychotherapy theory or opinions of mental health service professionals (such as clinicians and psychotherapists) or whether the usability of the app has been proved by peer-reviewed academic research? (Yes or no)

### Quality Appraisal of Apps

To evaluated the quality of the apps, we used the Mobile App Rating Scale (MARS), a validated scoring tool for assessing the quality of mHealth apps [23]. MARS has been used to evaluate the quality of different apps, such as apps for mental disorders [24,25], nutrition [26], drug-drug interaction checks [27,28], and chronic disease management [29-31]. MARS contains 23 items, including 4 objective quality subscales of engagement, functionality, aesthetics, and information quality and 1 subjective quality subscale. All items were rated on a 5-point Likert scale from 1 (inadequate) to 5 (excellent). We emphasized the objective quality of the apps, so the subjective quality subscale was excluded from the study. Before formal scoring, all reviewers evaluated the apps that provided only a single mental health service (excluded from the analysis) and discussed inconsistencies in and doubts regarding the results to ensure a unified understanding of MARS projects and standards. To fully experience the service provided by the apps, 2 independent reviewers downloaded and used each app for at least 15 min. The score of each subscale is calculated as the mean of the items in that subscale, and the total score is the mean of each subscale, which describes the overall quality of the app.

### Statistical Analysis

The quantitative variables of the MARS score in quality evaluation are described by the mean and SD. Classification variables, such as app characteristics, are described by frequency and percentage. To ensure the reliability of the quality assessment of 2 independent observers, the intragroup correlation coefficients (2-way random, mean measurement, and absolute consistency) were used to evaluate the consistency of commentators at the subscale and overall score level [32]. Pearson correlation coefficients were used to compare (1) the MARS total score and each subscale score, (2) the MARS total score and the user rating, and (3) the user rating and each subscale score. All statistical analyses were conducted using SPSS Statistics 25.

## Results

### App Selection

**Figure 1:**
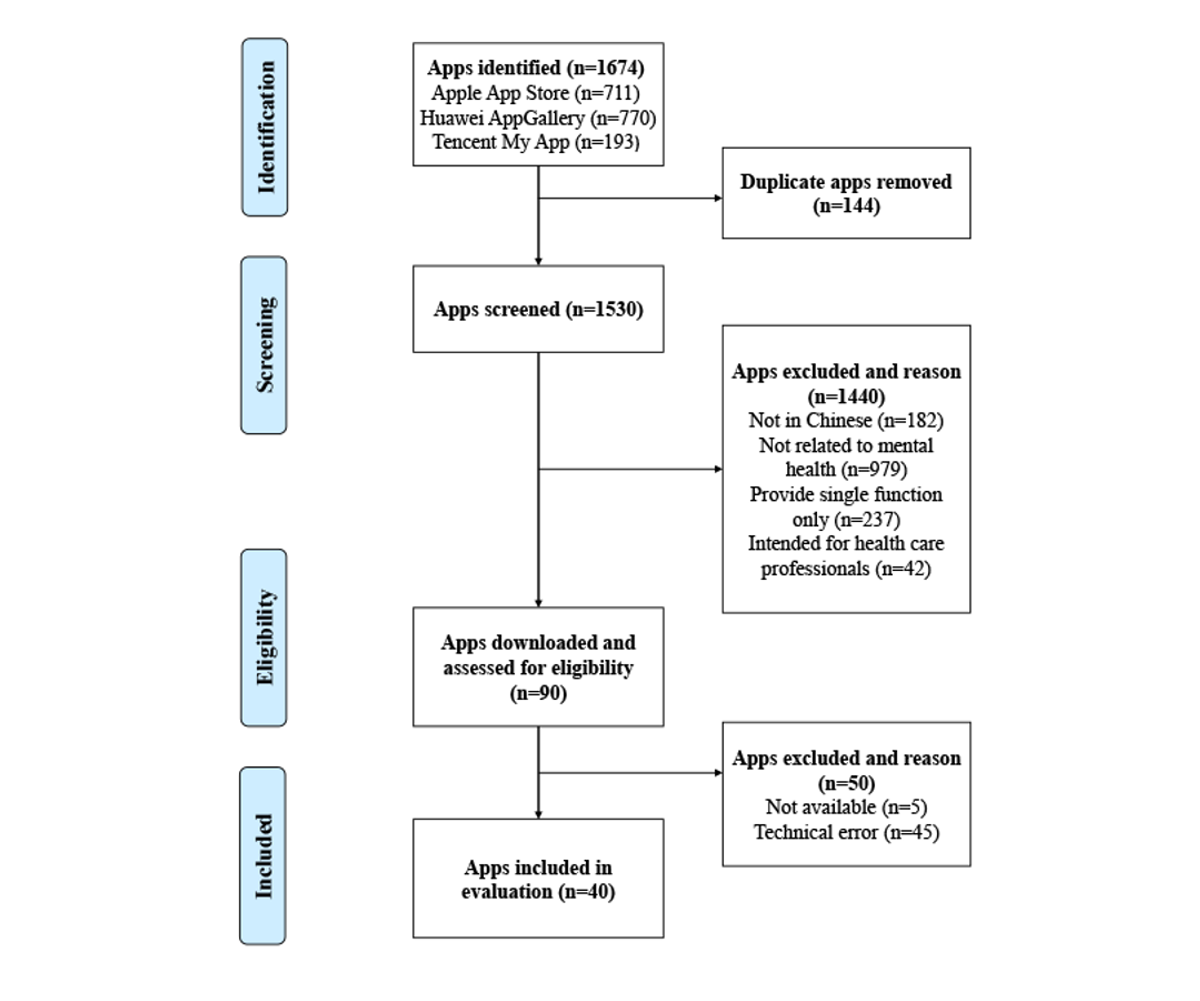
Flowchart for the systematic search and selection of apps.

A total of 1674 apps were identified through keyword retrieval (711 [42.47%] apps from Apple App Store, 770 [46.00%] apps from Huawei AppGallery, and 193 [11.53%] apps from Tencent My App). Combining the search results of the 3 app stores, 144 (8.6%) duplicate apps were excluded. A total of 1440 (86.02%) apps were excluded on the basis of the exclusion-inclusion criteria. The remaining 90 (5.38%) apps were downloaded onto the evaluation device for further evaluation. Of these 90 apps, 45 (50%) were excluded because of technical reasons (unable to download or use normally because of major technical reasons) and 5 (5.6%) were excluded because of the need to provide special authentication (employees/students). Finally, 40 (44.4%) apps were included in this study (Figure 1).

### General Characteristics

Of the 40 apps included, 31 (77.5%) are from Huawei AppGallery, 6 (15%) from Apple App Store, and 3 (7.5%) from Tencent My App. Most of the apps (35/40, 87.5%) are developed by companies (4 of them are companies mainly engaged in mental health services), 2 (5%) apps are from professional psychological counseling centers, and 3 (7.5%) apps are from individual developers. In addition, 2 of the 40 (5%) apps are specifically designed for adolescents or children. Furthermore, 21 (52.5%) were updated more than 1 month and less than 1 year ago, 15 (37.5%) were maintained within 1 month, and 4 (10%) were updated more than 1 year ago (Table 2).

**Table 2:** Flowchart for the systematic search and selection of apps (N=40).

Assessment measure			n (%)
**Platform**			
	Apple App Store	6 (15)	
	Huawei AppGallery	31 (77.5)	
	Tencent My App	3 (7.5)	
**Developer**			
	Commercial	35 (87.5)	
	Psychological service organization	2 (5)	
	Individual developer	3 (7.5)	
**Target user**			
	Children or adolescents	2 (5)	
	General public	38 (95)	
	Specific for women	0	
	Patients with mental health problems	0	
**Update time**			
	>1 year	4 (10)	
	>1 month and <1 year	21 (52.5)	
	<1 month	15 (37.5)	
**Privacy protection**			
	Privacy protection mentioned in the description	17 (42.5)	
	An obvious privacy protection logo	23 (57.5)	
	Relevant privacy protection regulations	33 (82.5)	
**Evidence-based and professional background^a^**			
	Proven psychotherapy theory	5 (12.5)	
	The opinions of mental health service professionals	14 (35)	
	Peer-reviewed academic research	1 (2.5)	
	Not mentioned	21 (52.5)	

^a^Some apps claim to be designed based on one or more scientific foundations.

### Privacy Protection

Of the 40 apps, 17 (42.5%) mentioned the protection of user privacy in the descriptive content of the app store. More than half of the apps (23/40, 57.5%) provide obvious identification during app use to remind users of privacy protection. Almost all apps (33/40, 82.5%) provide links in the app market interface or within the app to access the privacy policy. However, less than two-thirds of these 33 apps (n=21, 63.6%) display privacy policies before users log in to their accounts. All privacy policies explain how to collect user data, and almost all privacy policies (30/33, 90.9%) express how to share, transfer, and publicly disclose user data. Less than half of the apps explain how to store information (16/33, 48.5%) and how to use cookies and other similar technologies (15/33, 45.5%). Of the 33 privacy policies, 14 (42.4%) list the rights of users to manage personal data. For example, users have the right to delete or correct their data. Slightly more than half of the policies (19/33, 57.6%) explain the processing method of minor personal information. Finally, 18 of the 33 (54.5%) apps provide the contact information of the data protection officer that users can access when they have questions or opinions about the content of the privacy policy.

### Evidence-Based and Professional Background

In the description of the app market, 21 of the 40 (52.5%) apps do not mention that the app design specifies a relevant scientific basis. The remaining 19 (47.5%) apps claim to be designed on the basis of 1 or more scientific foundations: 14 (73.7%) apps are described as being designed according to the opinions of mental health professionals, such as clinicians, psychologists, and psychotherapists, while 5 (26.3%) apps claim to be designed using proven psychotherapy theories, such as cognitive behavioral therapy; of these 5 apps, only 1 (20%) claims that its usability has also been confirmed by peer-reviewed academic research.

### Functionality Review

**Figure 2:**
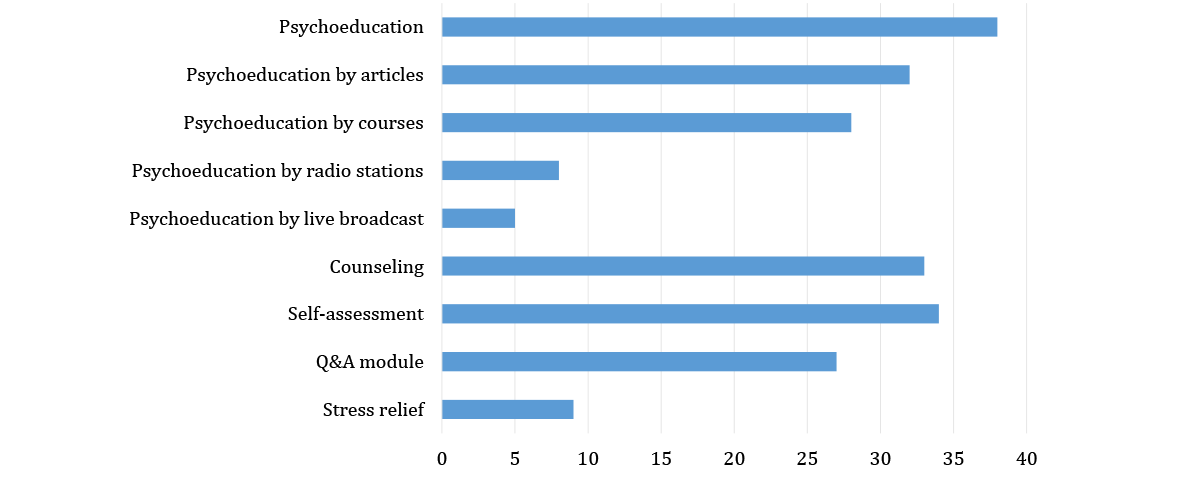
Functional review results of the multipurpose mental health apps. Q&A: question and answer.

Psychoeducation (38/40, 95%), counseling (33/40, 82.5%), and self-assessment (34/40, 85%) occur in more than three-quarters of the apps, the Q&A community (27/40, 67.5%) appears in about two-thirds of the apps, and stress relief modules (9/40, 22.5%) exist in less than a quarter of the apps (Figure 2). The most common combination in multipurpose apps is the combination of psychoeducation, counseling, self-assessment, and the Q&A community (14/40, 35%). The second is the combination of psychoeducation, counseling, and self-assessment (7/40, 17.5%).

**Figure 3:**
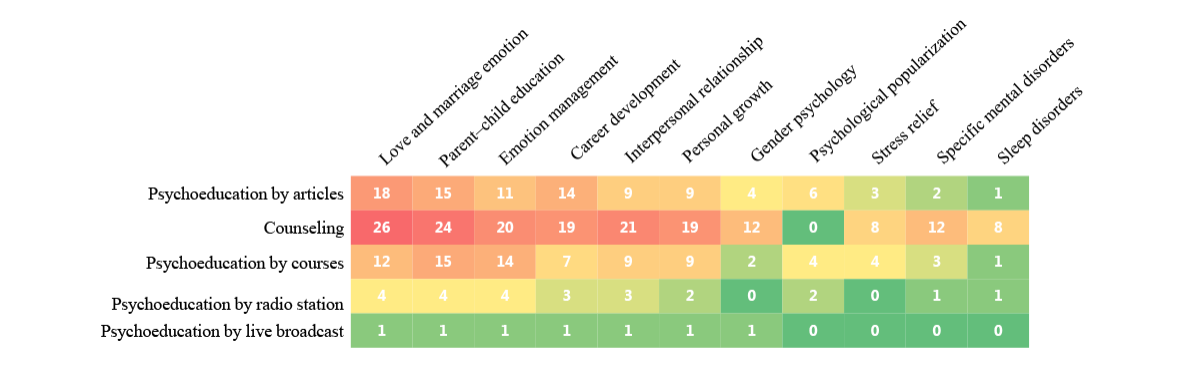
A heatmap of the topic type (top) for each function and the app function type. Note: numbers in white refer to the frequency of topic types involved in app functions. Warmer colors indicate higher counts.

Most of the applied psychological education, psychological counseling, and self-assessment function modules set up a topic classification in navigation. To understand the distribution of topics provided in the functional modules of the current apps, we created a heatmap, as shown in Figure 3. The most common themes are love and marriage emotion, parent-child education, and emotion management. The next most common themes are mental disorders in career development, interpersonal relationships, and personal growth.

### Psychoeducation

Almost all apps (38/40, 95%) provide psychological education intervention. The mental health education part of the apps is reflected by reading articles related to mental health (32/38, 84.2%), learning relevant courses (28/38, 73.7%), and obtaining relevant information through radio stations (8/38, 21.1%) or live broadcast (5/38, 13.2%).

Among them, the most common way is reading articles related to mental health. Most of these articles are originally created by the platform or psychological counselors, which have guiding and educational significance for the public. More than half of the apps (19/32, 59.4%) have classified the topics of articles to set columns. The main content of the columns includes love and marriage emotion (18/19, 94.7%), parent-child education (15/19, 78.9%), career development (14/19, 73.7%), and emotion management (11/19, 57.9%). Additionally, it also includes themes, such as interpersonal relationships (9/19, 47.4%), personal growth (9/19, 47.4%), gender psychology (4/19, 21.1%), and stress relief (6/19, 31.6%). However, only 2 of the 32 (6.3%) apps have set up columns for specific disorders, such as depression and anxiety, and 1 (3.1%) app has a column for sleep disorders. In addition, 2 (6.3%) apps have columns for students or teenagers, and 1 (3.1%) app set up a column with the rehabilitation story of psychological disorders as the main content.

Mental health–related courses record videos in the form of online education and invite mental health service professionals to present their professional knowledge, which covers common mental health knowledge, such as emotion management, love emotion, and psychological knowledge. Some of the app courses are free, while most charge a specific fee, ranging from as low as RMB 1.9 (US $0.3) to more than RMB 10,000 (US $1596). Only 6 of 28 (21.4%) apps offer completely free courses. Most other apps (22/28, 78.6%) provide free and paid courses at the same time, and users can choose based on their situation.

Radio and live broadcast are considered less psychological education ways. Mental health education provided using the radio station is mainly manifested in showing users past cases of mental health disorder adjustment or sharing common methods of mental health disorder adjustment. Live broadcast makes up for the shortcomings of the radio form. Users can directly contact consultants through a live broadcast, which strengthens the interaction between users and consultants.

### Counseling

Mental health counseling services are provided by mental health service providers, who help solve psychological problems and “heal the soul” through online listening and answering of questions. This functionality is provided in 33 of 40 (82.5%) of the apps. Particularly, the Xiaoxin Psychology app applies artificial intelligence technology to mental health services and provides online counseling services through intelligent robots. Additionally, almost all apps provide consulting services by psychological counselors, and users can select an appropriate provider by viewing the basic information about the psychological counseling provider or modifying the label. Common labels include professional qualification (31/33, 93.9%), areas of expertise (32/33, 97.0%), user evaluation (24/33, 72.7%), and service person-times (23/33, 69.7%). The professional qualification of psychological counselors is the key factor for users to choose from. However, only 19 of 33 (57.6%) apps clearly express the authenticity of professional qualification; 4 of these 19 (21.1%) apps provide evidence of professional psychological counselor qualification, such as a certificate photo or a certificate number. In addition, 15 of the 19 (78.9%) apps are guaranteed by the platform to ensure the authenticity of counselor data. All app downloads are free, but users are charged a specific consulting fee. The consultation cost varies depending on the time or number of times. Almost all consultants (30/33, 90.9%) use voice chat to communicate with consumers. Others provide consultation using text and pictures (21/33, 63.6), video communication (20/33, 60.6%), and offline face-to-face consultation (16/33, 48.5%).

### Self-Assessment

The most common function in the apps is psychological testing, accounting for 34 of 40 (85%) of the total. The apps provide some evidence-based or entertainment scales, and users can understand their mental health status through self-assessment of scale problems. Most apps (26/34, 76.5%) provide evidence-based scales, the most common of which include the Self-Rating Anxiety Scale (24/26, 92.3%) [33], the Self-Rating Depression Scale (22/26, 84.6%) [34], and the Symptom Checklist-90-Revised (14/26, 53.8%) [35]. Almost all apps (32/34, 94.1%) provide a scale for the nature of entertainment to attract users’ attention. These scales are developed by the app team or formed by a scale with unclear origin to evaluate the user’s emotion, personality, ability, sleep status, professional interest, and interpersonal status.

### Q&A Module

The Q&A community embodies the great advantages of mental health apps compared with traditional mental health services. Of the 40 apps, 27 (67.5%) provide this module. The Q&A community gathers other users and consultants on the same platform for rapid communication of mental health problems between users and between users and consultants, which is difficult to achieve by traditional psychological services. Users express their troubles, puzzles, or problems in the Q&A community, discuss and communicate with other users and consultants through Q&A feedback, solve problems, and gain knowledge.

### Stress Relief

The stress relief module regulates the user’s mood, improves the sleep state, and relieves the user’s psychological pressure through proven ways, such as meditation and audio decompression. Of the 40 apps, 9 (22.5%) provide functional modules for stress relief. Meditation is considered a popular intervention method to relieve stress. Of these 9 apps, 7 (77.8%) provide functional modules to assist meditation. The modules guide stress relief training based on mindfulness or breathing technology (7/7, 100%) and cognitive behavioral therapy (1/7, 14.3%) in the form of audio or video. Audio is the main medium to help users relieve pressure. In addition to the audio used for meditation, other types of audio include light music (4/9, 44.4%), autonomous sensor meridian response audio (1/9, 11.1%), and nature recording (4/9, 44.4%). Additionally, 4 (44.4%) apps specifically provide audio to help users sleep.

### MARS Evaluation

The overall MARS score showed high interreviewer reliability (intraclass correlation coefficient [ICC] 0.95, 95% CI 0.858-0.960). Simultaneously, all subscales also showed good consistency: engagement ICC 0.93 (95% CI 0.865-0.963), functionality ICC 0.71 (95% CI 0.462-0.847), aesthetics ICC 0.91 (95% CI 0.833-0.953), and information ICC 0.85 (95% CI 0.727-0.923).

The total average MARS score of all apps was 3.54 (SD 0.39), and the total score ranged from 2.96 (Enmasa Psychology) to 4.30 (Yi Psychology). The MARS score of 7 of 40 (17.5%) apps was ≥4. Furthermore, 30 of 40 (75%) apps had MARS scores ranging from 3.0 to 3.99. MARS scores of 3 of 40 (7.5%) apps ranged from 2.0 to 2.99. There were no apps with a score of <2.

**Figure 4:**
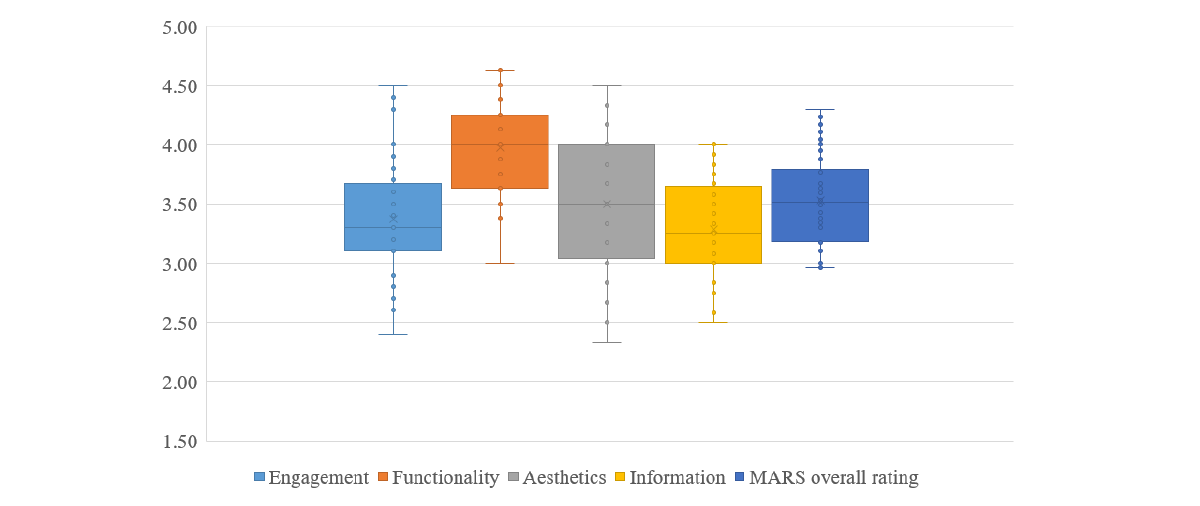
Graphical representation of the distribution of the MARS overall and subscale score. The median, the interquartile distance, and the range were given (N = 40). MARS: Mobile App Rating Scale.

The average scores of each subscale were as follows: information quality score=3.29 (SD 0.41), engagement quality score=3.37 (SD 0.51), aesthetic quality score=3.50 (SD 0.61), and functional quality score=3.97 (SD 0.37). The aesthetic quality score showed the largest span, with a minimum of 2.33 and a maximum of 4.50. The information quality part was the lowest, ranging from 2.50 to 4.00. The rating distribution of overall quality and 4 subscale dimensions is shown in Figure 4.

The overall MARS score was significantly positively correlated with the scores of each subscale (r=0.62-0.88, *P*<.001). However, the user rating of the app market was not significantly correlated with the total MARS score (r=0.17, *P*=.33) and the scores of various scales (r=0.05-0.22, *P*=.21–.77; Table 3).

Combined with the professional background of app development, the total average MARS score of the app described as designed according to the opinions of mental health professionals is 3.66. The total average score of apps claiming to use proven psychotherapy theory is 3.77. The only app that clearly states that its usability is confirmed by peer-reviewed academic research has a total MARS score of 3.95. The quality score for all of the above cases is higher than the overall average score for all apps. However, the total average score of apps without reference to app design involving relevant scientific basis is 3.40.

**Table 3:** Correlation between the Mobile App Rating Scale subscale and the overall score and the user star score.

Characteristic	Engagement, correlation (*P* value)	Functionality, correlation (*P* value)	Aesthetics, correlation (*P* value)	Information, correlation (*P* value)	Overall rating, correlation (*P* value)
Engagement	__^a^	__	__	__	__
Functionality	0.39 (.01)	__	__	__	__
Aesthetics	0.67 (<.001)	0.31 (.049)	__	__	__
Information	0.73 (<.001)	0.51 (.001)	0.65 (<.001)	__	__
Overall rating	0.88 (<.001)	0.62 (<.001)	0.86 (<.001)	0.88 (<.001)	__
User star rating^b^	0.22 (.21)	0.21 (.23)	0.08 (.64)	0.05 (.77)	0.17 (.33)

^a^Not applicable.

^b^Apps with zero user star ratings were excluded.

## Discussion

### Principal Findings

This study identified 40 multipurpose mental health apps, understood their main functional distribution, and evaluated their content and quality.

The evaluation of the professional background of app development provides an opportunity for app developers to improve the scientificity and accuracy of these apps. Among all included apps, only 2 (5%) are developed by professional organizations engaged in mental health services, while most of the apps (38/40, 95%) are developed by commercial companies or individuals without mental health–related backgrounds. Additionally, more than half of the app development background lacks the participation of professionals or scientific theories. In the description of the apps, only 1 (2.5%) clearly stated that its availability had been confirmed by peer-reviewed academic research. The absence of the development process in the app description may raise questions about the credibility of the apps [36]. Simultaneously, the lack of a professional development background may lead to an inappropriate final app, which is considered a potential threat to users. Previous evidence also emphasizes that the development process should include the participation of health care professionals and target users with regard to apps that provide health content and collect health data [37]. Therefore, these findings emphasize the need to take action to ensure the scientific quality of mental health apps, which will improve the reliability and quality of the content provided by the apps.

The in-depth analysis revealed that the most common functional combination of multipurpose mental health apps is psychological education, counseling, self-assessment, and the Q&A community. Only 1 (2.5%) app contains all functional modules, and its quality score is also good. In all apps, psychological education, self-assessment, and counseling occupy the main positions. However, the online consultation function of most apps is provided by professional mental health care personnel. Therefore, the problem of insufficient mental health service personnel still exists. However, we found in this search an app, Xiaoxin Psychology, that provides the possibility of solving this problem. Xiaoxin Psychology combines artificial intelligence with mental health to replace mental health service personnel. Although we have not found a test of the effectiveness of the app, studies have confirmed the effectiveness of evidence-based computerized interventions in alleviating anxiety and depression in adults [38].

Among the 40 apps identified, except “grape heart,” which is an app for children with autism, no app for a specific mental disorder was found. This is different from foreign mental health apps [39-41]. However, we found that the included apps classify the service theme and set up navigation in the menu for quick access. We observed that the obstacles of love and marriage emotion and parent-child education are the most common. This may be related to the traditional Chinese concept of paying attention to family emotion. COVID-19 has increased the demand for mental health services, but only 3 (7.5%) of all assessment apps have added psychological aid plates. This may be due to an untimely update of the current apps. App developers set content classification modules on the basis of current events and hot spots, which is a good way to attract new users and stabilize old users.

The 40 apps’ choice of target population also has specific characteristics. Only 2 (5%) of the apps are designed specifically for teenagers or children, and there are no apps designed for women. App developers prefer ordinary adult users, which may be because it is easier to obtain users and maintain the stability of users. However, adolescents are one of the most vulnerable to mental health problems [42]. They are often reluctant to seek professional help because of their sense of shame and tendency toward self-reliance [8]. The method of getting help based on a network provides a way to overcome these obstacles [43-45].

Although almost all apps provide privacy policies for the collection and use of users’ personal data, they lack detailed information about data storage, user management permissions, and the use of cookies. Moreover, in this evaluation, most apps lacked a description of the endpoint of data sharing, which is consistent with the results of previous studies [46]. The economic benefits of shared data promote the occurrence of such situations and pose a threat of user data disclosure [47]. However, personal health information is highly sensitive, and the disclosure of health information may cause varying degrees of negative effects and even death [48]. Additionally, people may refuse to use mHealth apps because of concerns about health data security and privacy [49,50]. Furthermore, concerns about privacy protection are exacerbated by people’s sense of shame about using mental health services [51-53]. However, the trust between app developers and users may be damaged by the lack of clarity of privacy policies, which results in the loss of potential long-term users [54]. Therefore, there is still a long way to go in terms of the compliance with privacy policy content and the pertinence to special types of apps (such as health).

The overall quality of the multipurpose mental health apps we reviewed is good. The MARS score ranges from 2.96 to 4.30. There is a gap in the quality of the apps, which is similar to that in previous studies [55]. However, the 3 (7.5%) apps with the highest MARS quality score also have the highest download frequency, indicating the attraction of high-quality apps to target users. Additionally, the apps show advantages in functional evaluation rather than the information part. This emphasizes that future app development should focus on improving the information quality of apps. Adding professional mental health care personnel to the app development process may be a feasible way. This will also provide a reference for users to evaluate the degree of expertise involved in the app development process before downloading [56]. In this study, apps with professional development backgrounds also reflected their advantages in quality scoring. The user star rating was not correlated with the total MARS quality score, which may indicate that there is a different structure between the user star rating and the app quality score. Similarly, in previous studies, it has been reported that app quality depends not only on the content but also on the function and design method of the content [57]. Furthermore, the star rating of the app market may involve the early version of the apps and cannot fully represent the current version, which may lead to distortion in the evaluation of the current version [58].

### Contribution

This study conducted a specific survey on the content and quality of multipurpose mental health apps in China based on a systematic and evidence-based approach. To the best of our knowledge, this is the first attempt to incorporate the professional background of app development and the user privacy protection policy into the evaluation of mental health apps in China. These findings will assist app developers in enhancing current apps or design new apps. Additionally, through the analysis of privacy policies, users can better understand the potential risks of providing information to service providers. Furthermore, we found that the combination of artificial intelligence and mental health may provide the possibility of solving the problem of insufficient mental health service personnel. This will be the direction of designing and creating apps in the future.

### Limitations and Future Work

There are some limitations to this study. We may have missed some apps. Keywords retrieval cannot exhaust all apps. Some apps that met the inclusion criteria were ignored because the title or description did not contain search criteria related to mental health. Moreover, the app market is constantly changing, new apps may be on the shelf at any time, and old apps may be deleted for various reasons. Although the research examines the apps’ emphasis on user privacy protection, we cannot verify whether the apps really implement privacy protection measures. Additionally, this study did not verify the scientificity of the content provided by the apps, and in-depth research will be continued in the future. Two researchers independently screened the eligibility of the apps, extracted the characteristics of the apps, and used MARS to evaluate the quality of the apps. The rater’s reliability is good or excellent, but if more researchers participate, the results may be more objective. Hence, we will consider increasing the number of researchers in follow-up studies. Furthermore, some of our findings may reveal the direction of such research in the future. We found some behavior change techniques aimed at improving users' mental health, such as meditation. Future research can further evaluate the quality and characteristics of behavior change techniques in these apps. In addition, this survey also identified some high-quality apps. Before widely recommending these apps, a further randomized controlled trial can be used to determine and compare their effectiveness.

### Conclusion

This study identified 40 multipurpose mental health apps, analyzed their main functional distribution, and evaluated their content and quality. These findings will assist app developers in enhancing current apps or design new apps. The quality of multipurpose mental health apps in China’s main app markets is generally good. Most apps provide rich functionality and classify the service theme to set up navigation in the menu for quick access. However, the lack of professional background in the app development process raises concerns about the scientificity of the apps. Furthermore, the privacy protection policy of the apps also needs to be described in more detail.

## Abbreviations

ICC: intraclass correlation coefficient
MARS: Mobile Application Rating Scale
mHealth: mobile health
Q&A: question and answer
